# The Practice of Percussion

**Published:** 1918-01-12

**Authors:** 


### THE PRACTICE OF PERCUSSION
### AS INFLUENCED BY THE NEWER METHODS.

### I. By A GENERAL PRACTITIONER.

THE tenor of the day's criticism and the comments upon the teaching value of lecturers should sound the death-knell of contributions to the medical press from those who make no pretence to be more than general practitioners. But some of us who are growing old at the work still cherish the memory of our teachers, and subscribe to the testimony made by Sir William Osler to Dr. Camac—"In the teacher I have always valued the message of the life above the message of the pen." The student's inheritance from his teacher is the recognition of the fact that the purpose of his life lies "not in achievement, but in faithful striving and the manner of his work."

If that be true, there should be few general practitioners who cannot add some useful points to the knowledge of everyday practice. Such contributions may be scraps, and may be dealt with as such; but even then they may find themselves in good company. Dr. Gee tells us that Auenbrugger, one of the earliest apostles of percussion, "was prepared to suffer from envy, hatred, and calumny; we know that he endured what is harder to bear, simple neglect." This same subject—percussion—is one of the things which we learn from our teachers, and we recognise it in their hands as a powerful diagnostic measure; yet in our own hands it is apt to fail, at least at times, to elicit the desired information. It is more often demonstrated than taught, and as a personal possession it is proportionate to the training of the ear and fingers. Even in our student days the absolute dulness of a pleural effusion was easy to recognise, but I do not think that I overstate the truth when I say that most of us found it more easy to follow auscultatory signs than to estimate a slight comparative dulness. In early practice, contact with one's fellow-practitioners emphasised this point. An opinion upon deficient tone was sometimes met by an expression of doubt, at other times by a significant silence, although we were aware of the importance which our skilled leaders would have assigned to it. No one was more cognisant of the difficulty than myself, and I sought eagerly for a solution of it. Percussion is a matter of tone, and the appreciative ear is not the possession of everyone. But the first element in music is time, and the varieties in tone become more obvious when they are adapted to time. The skilled violinist can tune his strings without the added element of time, but he seldom ventures an entry upon the piece of music which he is about to play without a preliminary canter in musical time. Why should it not be the same with percussion when we seek its finer qualities? Help in this direction will be all the more needful when members of our profession return to work after having spent months or years within sound of the guns. When in doubt as to a slight comparative dulness try the effect of adding a marching or waltzing time to the percussing finger, and pass it from one to the other area of the parts in question. Every chest offers the opportunity, for the tone of the right apex is normally less resonant than that of the left. If they are no more than equal, the slightest change in the intensity or character of the respiratory murmur on the left side attains a more grave significance. And what is true of the anterior apex is still more valuable when applied to the posterior second apex. The late Dr. C. J. B. Williams was the first to question the common belief that phthisis most frequently began at the left apex; but he maintained that when it did begin on the left side it made more rapid progress. Other writers of more recent date have shown that the rapidly progressing cases are those in which the second left posterior apex falls a victim to infection, and that the infection in this situation spreads rapidly down to the base of the lung.

A glance at the *x*-ray photograph of the bronchial tree in Sir Douglas Powell and Dr. Hartley's book on "Diseases of the Lungs" furnishes a reasonable explanation. The large bronchial tube and its almost vertical descent favour the spread of infection. It is important to remember also the effect which the physical condition has upon the normal auscultatory sounds, and this consideration emphasises the need for a careful and discriminating percussion, for by this solidification may be recognised before the T.B. is found in the sputum. There is another point upon which percussion offers evidence. It is unlikely that all the inhaled infection settles in one apex, and it is certain that wherever it does settle it provokes protective resistance in the form of a greater or lesser degree of congestive or inflammatory change. Careful percussion will show dulness sometimes greater on the right side and at other times greater on the left in the same patient. My earlier recognitions of this led me to doubt the accuracy of my notes, but careful watching in fresh cases has proved it to be true. Some infected spots lost all their signs so completely that men whom I am proud to recognise as my teachers overlooked them, and yet the disease reappeared in them in later years.

The general practitioner's note-book should be a record of facts, rather than a mere nominal diagnosis. The history of serious illness should not be omitted, but the record of slight signs which one discovers in patients calling at the consulting-room is the sum of the patient's handicap. When grave disease does occur, a revision of these recorded facts indicates the direction of probable failure and favours successful treatment. The records may be scrappy, but they should not be scrapped. We recognise their value more and more as the years go by, and this in regard both to the individual principally concerned and to members of the next generation.

Beyond the addition of time towards the elicitation of tone in the act of percussion it is well to

**THE PRACTICE OF PERCUSSION**—(*continued*).

note that the finger which is applied to the chest should not be pressed sufficiently hard to limit the respiratory movements, but only firmly enough to secure close contact with the chest wall. The strength of the stroke has some influence in securing a more or less deep response, but here, as in abdominal palpation, the gentler touch is the more reliable.

The authority for these notes should be sought for in their confirmation after due trial. It may be a long process, but if I have made the points clear it will be less long to my fellow-workers than it has been to myself.

### II. By A HOSPITAL PHYSICIAN.

No one interested in physical examination—and this, after all, is one of the main agencies in the detection of disease—will speak lightly of any attempt to improve the practice of percussion. The art is a difficult one, and anything like an approach to its full achievement is only possible by much practice and by the use of a right method. It may be quite a fair thing to say that it receives less attention to-day than in an earlier time, and less, too, than its value deserves. For this there is a very obvious reason. Physical examination has not lost its value, but it has now to suffer competitors or rivals, whereas formerly, as an agency for the discovery of the objective evidences of organic change, it stood alone, or almost alone. Under the influence of this competition it is almost inevitable it should incur comparative neglect. Attention which has to be divided cannot have the degree of value which concentration or restriction can boast. Moreover, certain of the newer methods have an appearance of ease and certainty which the older clinical proceedings cannot claim. To cultivate the ability to detect slight changes in the percussion note, or to appreciate and rightly to interpret auscultatory sounds, means a long and decidedly tedious education, while it must be admitted also that there are occasions on which competent men will differ among themselves both as to the facts and as to their precise significance. On the other hand, to observe the picture of the organs of the chest as these can be exhibited on the screen by the agency of the *x*-rays offers itself as a simple proceeding and one which has both an entertaining quality and a clear-cut result. The student has but to use his eyes and he sees as it were the very facts themselves. This at least is the immediate and obvious view of the matter; the difficulties and limitations come into view later on. Similarly, the staining and detection of tubercle bacilli in the sputa appears as a simple pleasing enterprise, and it offers, in one direction or the other, a quite definite result. In both of these examples there is seen a sharp contrast to the slow and dull discipline which percussion and auscultation demand. Little wonder therefore that the modern student takes the easier, more pleasant, and, as it seems to him, the more certain way, and that he shirks the sustained and depressing training which the older methods require. Probably it is not unfair to suggest that even the teachers do not wholly escape the temptation to press less forcibly than used to be the case the importance of percussion, auscultation and the rest. The teaching of these is hardly less tedious than the learning of them. Nor do these remarks restrict themselves to the examination of the thorax. Equally in the case of the abdomen there is the vision revealed by the *x*-rays, aided or not by the bismuth meal. Further, here is always at hand the ready surgeon who will cut the Gordian knot of a difficult diagnosis by the quick and certain sword of an exploratory operation. Why remain in doubt when it is so easy to open the abdomen and see the direct facts? No one, we think, will deny that influences of the order here suggested are active in modern medical education. That the newer methods have their value is beyond question, and they are to be welcomed and encouraged. But they ought to be welcomed and encouraged not as substitutes for the older practices but as additions to these. There is a danger lest the student comes to exalt unduly the pictorial and dramatic qualities of the skiagram and the staining agent, and in so doing neglects the more drab and dull proceedings of physical examination. The appeal of the one is most engaging and seems to promise absolute certainties. The other means a much more tedious exercise and, at least sometimes, leaves even at the end a margin of doubt. Is it a matter for wonder that the student will cleave to the one rather than to the other unless under firm and wise direction he is led to recognise that each has its own sphere and that neither can be neglected except at the peril of inefficiency?

One thing which has to be remembered in this connection is that the conditions of the medical school are not exactly the same as those of family practice. In the former, special apparatus and skilled manipulators are always at hand, or can easily be secured. But in the outer world these things cost money, and, relative to the circumstances of many patients, a good deal of money. An *x*-ray machine can hardly be carried from house to house, and chemical analyses mean time and judgment as well as equipment. If these methods are to be the principal guides to the practitioner, he will meet with many circumstances in which they are simply not available. To depend upon them means to depend upon help which cannot be secured. Again, a frequent proposal of an exploratory operation will in private practice be found to be much less popular than in hospital life, and, offered as a means of diagnosis merely, it will hardly excite the confidence of patients who are more anxious to get well than to fill an accurate place in the ordered classification of disease. Still further, experience gradually reveals that even skiagrams do not always tell the truth, or not at least the whole truth, and that while stains have their values the patient himself is not without importance in the diagnostic debate. By all means let all agents be employed which can

**THE PRACTICE OF PERCUSSION**—(*continued*).

possibly give help either in the interpretation of symptoms or in their cure. But it will be a bad day for medical practice when the tried and justified scheme of physical examination of the patient is neglected for ambitions which are more showy, which admittedly have a decided value, but which are less generally available and are also less free from ambiguities than their superficial characters propose. The one task must be done, but not less certainly the other must not be left undone.

If what is here said about physical examination in general is true, the principle applies with special force to percussion, which is certainly far and away the most difficult of the physical methods to practise with success. Doubtless there are degrees of excellence even when equal industry is engaged. Not everyone can boast the perfect appreciation of sound in its various characters and degrees, and original equipment is a factor as well as patience and culture and perseverance. Still, given these latter elements, everyone may develop and improve his capacity, and the reward will not be a slight one. Let us, however, be perfectly frank and say that of all the methods of examination in no other is there such an opportunity for self-deception as in percussion. Something more than goodwill is necessary, something more even than mechanical dexterity and the attuned ear. Mental honesty is an essential part of the performance, and a foregone conclusion or even an encouraged suspicion is fatal to success. Let this part of the equipment be wanting and the slope is a slippery one, for the performer is apt to become the victim of a fixed delusion and to develop conclusions which rest purely on an imaginative basis. As for the teaching of percussion, it can no more be conveyed by words than can instruction in violin-playing. Actual demonstration and practice are the only avenues to success, and the practice must be continued under the condition just defined. If a criticism of individual performers be allowed, there is a temptation to say that some cultivate an unduly heavy stroke, and not a few are too hurried in their attack. The lightest stroke which will evoke resonance is generally the most fruitful, and after each blow time should be given for the ear to judge the value of the response. To rush rapidly from point to point is likely to produce confusion, and each effort should be judged before the next is attempted.

